# Immunoblotting Analysis of Fruit Proteins in Mexican Pediatric Patients Suggests the Existence of New Allergens

**DOI:** 10.3390/diseases13090284

**Published:** 2025-09-01

**Authors:** Angélica Torres-Arroyo, Maidelen Suárez-Gutiérrez, Andrea Iglesias-Amaya, Aramiz López-Durán, Luisa Díaz-García, Horacio Reyes-Vivas, David Alejandro Mendoza-Hernández

**Affiliations:** 1 Laboratorio de Bioquímica-Genética, Instituto Nacional de Pediatría, Av. Insurgentes Sur No. 3700-C, Col. Insurgentes Cuicuilco, Alcaldía Coyoacán, Mexico City C.P. 04530, Mexico; angelica.tarroyo@gmail.com (A.T.-A.); maide15@hotmail.es (M.S.-G.); 2 División de Ciencias Biológicas y de la Salud, Universidad Autónoma Metropolitana, Campus Iztapalapa, Mexico City C.P. 09340, Mexico; 3 Servicio de Alergia, Instituto Nacional de Pediatría, Av. Insurgentes Sur No. 3700-C, Col. Insurgentes Cuicuilco, Alcaldía Coyoacán, Mexico City C.P. 04530, Mexico; andrea.unam95@gmail.com; 4 Servicio de Ortopedia, Instituto Nacional de Pediatría, Av. Insurgentes Sur No. 3700-C, Col. Insurgentes Cuicuilco, Alcaldía Coyoacán, Mexico City C.P. 04530, Mexico; aramizl@hotmail.com; 5 Departamento de Metodología de la Investigación, Instituto Nacional de Pediatría, Av. Insurgentes Sur No. 3700-C, Col. Insurgentes Cuicuilco, Alcaldía Coyoacán, Mexico City C.P. 04530, Mexico; luisadiazg@gmail.com

**Keywords:** immunoproteomics, food allergy, IgE-mediated allergy, pollen-food allergy syndrome, western-blot, oral allergy syndrome

## Abstract

Background: Food allergies are chronic diseases that compromise quality of life and can be potentially fatal due to anaphylaxis. The WHO estimates a 1–11% global prevalence, which has been increasing in recent years. They are considered, along with obesity, to be the two noninfectious pandemics. The WHO databases (WHO/IUIS) contain 403 food allergens, most of which have been reported from North America (Canada and the USA), Europe, and Asia, while reports of allergens from Latin America are scarce. Allergies have population and geographical specificities; therefore, identifying the main clinically relevant food allergens and potential new, undescribed components affecting Latin America is essential. This work aims to contribute to this field. Methods: we gathered data from 16 allergic Mexican pediatric patients to fruits from the Rosaceae (pear and peach) and Musaceae (banana) families, as well as an allergic adult to Lauraceae (avocado). These fruits are prevalent allergens in Latin America. Results: the data suggest that patients reacted to 20 different allergenic proteins reported in different allergen databases. Furthermore, we identified 16 previously unreported immunoreactive proteins, suggesting their potential role as new allergens. Conclusion: this preliminary work is particularly relevant, as it can influence the specific diagnosis of allergens most frequently affecting the pediatric population.

## 1. Introduction

The World Health Organization (WHO) states that allergies, together with obesity, are currently the two noninfectious pandemics; allergies affect nearly 150 million people and have a significant impact on the quality of life of patients [1]. Inadequate and delayed care for allergies can lead to a chronic pathological condition and even anaphylaxis, which can be potentially deadly. The clinical manifestations of allergies are varied and can be classified into systemic categories, such as respiratory, cutaneous, gastrointestinal, among others. Allergies are immune disorders and may include antibody-mediated, cell-mediated, or both mechanisms. The global prevalence of allergies is around 25%, with a tendency to increase in developed countries [1]. Regarding food allergies, the estimated prevalence ranged from 1.1% to 10.8%, depending on the country and the age of the patient [2]. There is a worldwide conglomerate called “the eight main groups of food allergens” that includes eggs, peanuts, walnuts, fish and shellfish, wheat, soy, sesame, and cow’s milk. Their allergenicity frequencies vary depending on the population and geographical region [3]. This indicates that the clinical relevance of allergens changes depending on the specific area of the planet. For instance, in Europe, the prevalence of egg allergy is 1.7%, whereas in Asia, the Middle East, and Africa, it ranges from 3.0 to 3.3% [4]; in contrast the prevalence of peanut allergy ranged from 2% to 11%, whereas in Canada, the value decreases to 1% [5]. In Latin America, there are few epidemiological studies on food allergies [6,7], but analyses in Colombia and Mexico, suggests that the prevalence in adults ranges from 10% to 30% [8,9]. On the other hand, studies in El Salvador, Argentina, Colombia, Chile, and Brazil indicate that the prevalence in the pediatric population ranges from 0.4% to 15% [10,11]. However, it is crucial to highlight that the data in such studies are primarily derived from parental surveys or self-report surveys, which do not necessarily delineate between clinically relevant food allergies. This circumstance must be considered, as the data may contain significant biases.

In food allergy, it is essential to note the presence of a condition resulting from previous sensitization to allergens from pollens, generating cross-reactivity reactions with fruits and vegetables, known as pollen-food allergy syndrome (PFAS) [12]. One of the recurring symptoms of PFAS, as well as other cross-reactions like latex with proteins from tropical plants [13], is oral allergy syndrome (OAS), characterized by the immediate appearance of oropharyngeal symptoms after consuming an allergenic food [14]; two percent of patients with OAS experience anaphylaxis [15,16]. Concerning Latin America, Mexico has conducted the most significant number of studies on PFAS and OAS. Studies in adult patients have shown that the primary triggering food allergies are shrimp, avocado, cow’s milk, almond, and fruits of the Rosaceae family (apple, peach, and pear), as well as the Musaceae (banana) and Actinidiaceae (kiwi) families [17,18,19,20]. In Mexican pediatric population, some of the principal allergenic foods are fruits from the Rosaceae (apple, pear, and peach) and Musaceae (banana) families; Rosaceae fruits are also the most frequent cause of allergy in Southern Europe, unlike in regions such as Asia or North America (USA and Canada), where fruits from Actinidiaceae, Musaceae, and Anacardiaceae families are the most important allergens [21,22,23]. We focused on this work on immunoglobulin E (IgE)-mediated food allergies, highlighting the essential role of proteins as allergenic components. The allergen databases, such as WHO/IUIS (allergen.org), COMPARE (comparedatabase.org), and Allergome (allergome.org), primarily contain information from studies conducted in North America (Canada and the United States), Europe, and Asia. In contrast, the Latin American contribution to the understanding of allergens at the molecular level is limited. This is critical since the clinical relevance of allergens depends on the geographic region, being essential to determine whether the allergens reported in these databases are representative of Latin America or if there are unidentified components that should be considered for the diagnosis and possible treatment of this disease. This information is crucial for assisting clinicians in the component-resolved diagnosis and will enable personalized medicine and potential immunotherapy.

To address this information gap, we conducted immunological studies on protein extracts from fruits of the Rosaceae (pear and peach) and Musaceae (banana) families using samples from Mexican pediatric patients with IgE-mediated allergy to these fruits. We also present results from a patient allergic to avocado (*Persea americana*, family Lauraceae) who experienced anaphylaxis due to accidental consumption. The databases from WHO/IUIS, Allergome, and COMPARE reports four allergens from pear with molecular weight (MW) ranges of 9–34 kDa. For peach, eight allergens have been reported with MW ranges of 7–62 kDa, whereas banana contains seven allergens with MW between 9 and 55 kDa. In contrast, the databases describe only two allergens for avocado, with MWs of 14 and 32 kDa; however, four additional new allergens have been identified in 2012 [24].

Our preliminary findings reveal reactivity against 20 known allergens, based on the MW ranges database. However, the results also provided evidence of immunoreactivity against 16 bands whose MWs do not correspond with some components reported in the database. Therefore, the results suggest the presence of possible new allergenic molecular components.

## 2. Materials and Methods

### 2.1. Study Design

We assessed a clinical, prospective, transversal and observational trial. To maintain patient confidentiality, a numerical code was created for the samples. All parents or guardians of the participating patients signed letters of consent and informed assent. A copy of these trades is included in the Supplementary Material. This study was approved by the Ethics, Biosafety, and Research Committees of the INP (Registration No. INP-035/2018).

#### 2.1.1. Patient Group

From 2018–2019, 16 pediatric patients (aged 6–16 years) with a pollen-food allergy syndrome (PFAS) diagnosis were recruited. The patients presented clinical manifestations related to consuming any of these three fruits or their combination: peach, pear, or banana. The inclusion criteria considered eating habits and the parent’s medical history, which was relevant to the diagnosis. In addition, we carried out a systematic study of immediate symptoms due to the ingestion of fruits. All patients included in the study were confirmed to have fruit-induced symptoms by open oral challenge test (OFC), a dermatological test (SPT), or both. For SPT, the method was performed with natural fresh food in a prick-prick version, using a skin and pulp mixture; the positive and negative controls for this last assay were histamine (10 mg/mL) and standard saline, respectively. The utilization of fresh fruits in this study is relevant, considering the importance of the freshness of antigenic food components in obtaining reliable results [25]. The SPT results were determined after 15 min of exposure, with a papule size ≅ 3 mm larger than that of the negative control being considered a positive test [26]. Information on patients’ sensitivity to *Quercus*, *Alnus*, or *Betula* pollen was also included. Additionally, we included a sample of a 28-year-old patient with an avocado allergy who presented anaphylaxis immediately after accidental ingestion, with respiratory, cardiovascular, and neurological clinical manifestations. The exclusion criteria for the study were patients treated with immunosuppressants such as steroids, chronic diseases, or conditions that could confound the diagnosis or the interpretation of the diagnostic profile.

#### 2.1.2. Control Group

We recruited eight patients (five girls) from the Orthopedic Service. Their ages ranged from 7 months to 16 years, with a median of 9 years and one month. Selected patients were under treatment due to fractures, sprains, or hip dysplasia. Medical records confirmed that both the patients and their families had no allergies, and any infectious or immunological diseases that could interfere with the study were excluded.

### 2.2. Biological Samples

Approximately 2 mL of peripheral blood was taken from each patient. The blood was centrifuged at 530× *g*/20 min at 25 °C to separate the cell package. The resulting supernatant was collected and centrifuged at 12,000× *g*/20 min at 4 °C. The supernatant was recovered and divided into 200 µL aliquots to be stored at −70 °C until use.

### 2.3. Protein Extraction

The protein was extracted from the four foods (peach, pear, banana, and avocado) via the technique described previously [27]. Briefly, 100 g of fruit pulp was cut into pieces and flash-frozen with liquid N_2_. The pieces were pulverized using a blender with a steel cup. The pulverized sample was solubilized in 200 mL of extraction buffer containing 30% sucrose, 5% 2-mercaptoethanol, 2% sodium dodecyl sulfate, one tables of complete protease inhibitors (Roche), and 0.1 M Trizma-base, pH 8.0. The sample was incubated overnight with shaking at 4 °C, and subsequently, 200 mL of Tris-saturated phenol (pH 8.0) was added. The sample was incubated overnight with shaking at 4 °C. Next, the mixture was centrifuged at 3220× *g*/50 min at 4 °C, and the organic phase was recovered; subsequently, 1 L of 0.1 M ammonium acetate in MeOH was added. The sample was incubated overnight at 4 °C with shaking; then, it was centrifuged again, and the buttons were recovered, pooled, and washed with cold MeOH. The protein extract was then washed with cold acetone. Finally, it was dried and stored at −70 °C until use. For the assays, the buttons were solubilized in a solution with 8 M urea and 2 M thiourea. The protein concentration was determined via the Lowry-TCA method [27,28]. Tris-saturated phenol was prepared in-house. Briefly, inside a fume hood, 500 mL of molecular grade phenol was melting in a 60 °C water bath. Then, 8-hydroxiquinoline was added to a final concentration of 0.1%. After that, 200 mL of 0.5 M Trizma-base (pH 8.0) was added and shaken with a magnetic stirrer for 30 min at 25 °C. The aqueous phase was discarded, and 100 mL of 0.1 M Trizma-base (pH 8.0) was added, followed by shaking for 30 min at 25 °C. The aqueous phase was again discarded, and 100 mL of 0.1 M Trizma-base (pH 8.0) was added and shaken for 30 min/25 °C. This procedure was repeated until the aqueous phase had a pH of 8.0. Then, the solution was stored in the dark at 4 °C.

### 2.4. Serological Test

Total IgE levels were determined by ELISA using the conditions described previously [27].

### 2.5. SDS-PAGE Assays

SDS-PAGE assays were performed using the Mini-PROTEAN Tetra cell system with 1.0 mm thick spacers (Bio-Rad, Dreieich, Germany). All gels were prepared and packaged in-house and contained 12% acrylamide supplemented with 10% glycerol and under reducing conditions [27,29]. Samples were run in duplicate on the same gel; one half of the samples was stained with colloidal Coomassie brilliant blue stain [30] and then photodocumented. Photodocumentation of the gels was performed via a ChemiDoc system (Bio-Rad). Alternatively, the other half of gels was transferred to PVDF membranes and subsequent Western blot assays.

### 2.6. Western Blot Assays

The trials were carried out under the methodological conditions described previously [27,31]. Briefly, from the SDS-PAGE assays, food proteins were transferred to PVDF membranes via a semidry system (Bio-Rad Trans-Blot semidry cell). Transferring conditions were as follows. The membranes were run at 271 mAmp/30 min using transferring buffer containing 0.25 M Trizma, pH 8.3, 20% ethanol, and 0.192 M glycine. Then, the membranes were washed three times for five minutes with PBS buffer supplemented with 0.05% Tween 20 (PBS-Tween 20 buffer) and then, washed two times only with PBS buffer. Then, they were blocked for two hours at 25 °C with gentle agitation using PBS-Tween 20 buffer supplemented with 5% polyvinylpyrrolidone and 20% methanol. The membranes were washed three times for five minutes with PBS-Tween 20 buffer, and then washed two times with PBS buffer. After that, the membranes were incubated overnight at 4 °C with a serum pool from patients or controls at a 1:100 dilution in PBS-Tween 20 buffer with gentle agitation. The membranes were washed three times for five minutes with PBS-Tween 20 buffer, and then washed with PBS, and incubated for two hours at 25 °C with HRP-labeled anti-human IgE conjugate (1:8000, Abcam Cat. No. Ab99806) diluted in PBS-Tween 20 buffer, and with gentle agitation. After washing the membranes to remove the non-bound conjugate, the proteins were detected by chemiluminescence using the Immobilon Western kit (Merck-Millipore Cat. No. WBKLS0500, Darmstadt, Germany) via a ChemiDoc system (Bio-Rad). These experiments were performed in triplicate.

### 2.7. Determination of Allergenic Proteins in Protein Extracts

The molecular weights of the detected immunoreactive proteins were calculated by comparing the Western blot and SDS-PAGE results stained with colloidal Coomassie brilliant blue stain. The precise alignment between the immunoblots and gels was conducted as follows. Samples were run in duplicate on the same gel; one half of the samples was stained, while the other half was transferred to PVDF membranes for Western blotting. The PVDF membranes were cut to the same size as the gels, aligning them by the bottom region. After transferring the proteins, we stained the membranes with Ponceau red to verify the successful transfer. Finally, after digitizing the Western blot images, we compared the band patterns from the membranes with those observed in the stained gels; in some cases, we used the most abundant proteins detected by the IgE as references. This information was used to search in the database of WHO/IUIS (allergen.org), Allergome (allergome.org), and COMPARE (comparedatabase.org) for allergens matching the identified molecular weights of the extracts in peach (*Prunus persica*), pear (*Pyrus communis*), banana (*Musa paradisiaca*), and avocado (*Persea Americana*).

### 2.8. Statistical Analysis

The total IgE data are presented as the means ± SDs. The odds ratio values were determined using the chi-square test, where a *p*-value <0.05 was considered statistically significant.

## 3. Results

### 3.1. Characterization of Patients

We included 16 pediatric patients (nine males and seven females) with a fruit allergy diagnosis and pollen-fruit allergy syndrome (PFAS). Their ages ranged from 3 to 16 years old (median of 11 years and 3 months ± 3 years and 8 months). All patients confirmed the allergy diagnosis through skin-prick test (SPT), oral challenge test (OCT), or both. The clinical data (Table 1) indicated sensitizations to peach in 75% (12/16) of cases, banana in 37.5% (6/16), and pear in 31.3% (5/16). Sensitization to one fruit was observed in 62.5% (10/16) of patients, followed by 31.3% (5/16) for two fruits, whereas 6.3% (1/16) showed allergy to three fruits. The total IgE concentrations ranged from 13 to 325 IU/mL.

Pollen sensitization frequencies by *Quercus*, *Betula*, and *Alnus* were quantified through SPT, showing *Quercus* as the main sensitizer with 75% (12/16) of cases, followed by *Betula* with 62.5% (10/16), and *Alnus* with 43.8% (7/16). In addition, we included a 28-years-old patient with an anaphylaxis history by accidental avocado ingestion, exhibiting three anaphylactic events after immediate avocado intake. Additionally, the patients experienced an anaphylactic event due to SARS-CoV-2 vaccine administration; however, their total IgE serum value was only 24 IU/mL.

**Figure 1:**
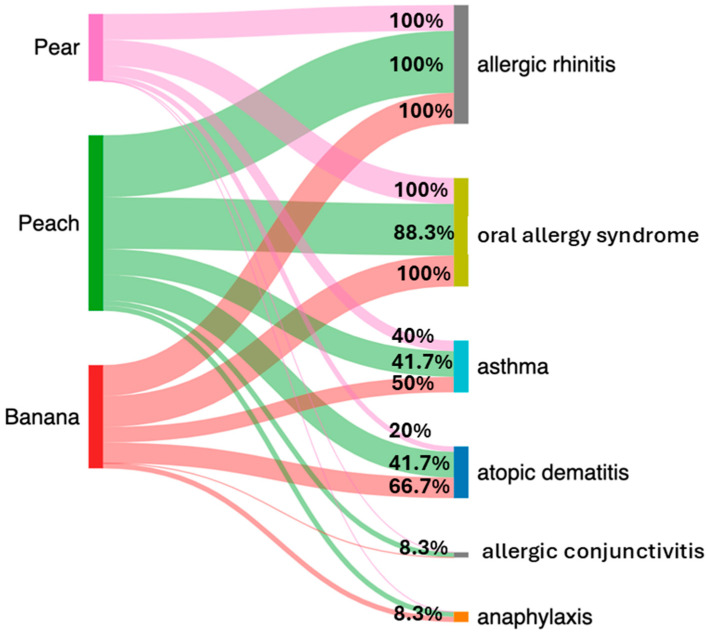
Sankey Diagram showing the clinical manifestation frequencies associated with each fruit. The percentage value represents the frequencies for each fruit group in the respective manifestation.

The frequencies of clinical manifestation associated with each fruit were as follows (Figure 1). Allergic rhinitis was detected in 100% of patients from the three groups. The OAS frequency was 100% for pear (5/5) and banana (6/6), while the peach group exhibited 83.3% (10/12). Asthma frequencies were 50% (3/6) for banana, 41.7% (5/12) for peach, and 40% (2/5) for the pear group. Atopic dermatitis values were 66.7% (4/6) for banana, followed by 41.7% (5/12) for peach, and 20% (1/5) for pear. For allergic conjunctivitis, it was observed only in the peach group with a frequency of 8.3% (1/12). Finally, anaphylaxis was observed in a patient with both banana and peach allergy, with a frequency value of 8.3% (1/12).

Notably, we identified two odds ratio (OR) values in the banana group. The first analysis showed an OR of 1.5 (95% confidence interval [CI]; 0.19–11.53) for banana and asthma, whereas the second analysis showed an OR of 4.66 (95% CI; 0.53–40.88) for banana and atopic dermatitis.

### 3.2. Extraction of Fruit Proteins

**Figure 2:**
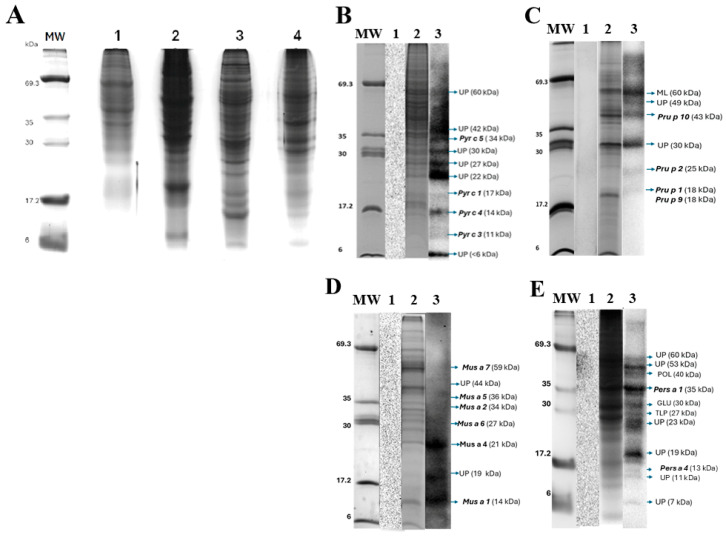
SDS-PAGE assays and WB assays of immunoreactive proteins from fruits. (**A**) Food extracts stained with colloidal Coomassie blue brilliant stain. MW: molecular weight markers; Lane 1: pear extract (*Pyrus communis*); Lane 2: peach extract (*Prunnus persica*); Lane 3: banana extract (*Musa paradisiaca*); Lane 4: avocado extract (*Persea americana*). All lanes contained 40 µg of applied protein; (**B**) Pear; (**C**) Peach; (**D**) Banana; (**E**) Avocado. Lane 1 of all panels corresponds to a representative WB assay of the serum sample from non-allergic patients (Controls). Lane 2 of all panels shows a representative SDS-PAGE of protein extracts. Lane 3 of all panels shows a representative WB assay of the serum sample of allergic patients. UP: Unknown protein. ML: Mandelonitrile lyase. PROF: Profilin. TLP: Thaumatin-like protein. POL: Polygalacturonase. GLU: Glucanase.

We evaluated the quality of fruit extracts (pear, peach, banana, and avocado) through SDS-PAGE (Figure 2A). Our extraction method proved effective in obtaining a significant number of proteins with a broad molecular weight distribution without any apparent degradation. The average extraction yields per 100 g of fruit were 32 mg for pear, 42 mg for peach, 94 mg for banana, and 450 mg for avocado. These values were consistent across different batches throughout the year. No batch effects in protein extraction or immunoblotting were observed during the period of study. Notably, our homemade gels provided sufficient resolution for determining the relative molecular weight of each band.

### 3.3. Allergens Identified by Molecular Weight Comparison

For the SDS-PAGE assays of the extracts of each food, gels were made in parallel for staining with colloidal Coomassie brilliant blue stain or Western blotting. In these trials, we used a pool of sera from allergic patients to determine specific IgE reactions against the proteins in each extract.

#### 3.3.1. Pear (*Pyrus communis*)

The results revealed IgE-mediated reactions against ten different proteins (Figure 2B). The immunoreactive proteins had an MW range of 6–60 kDa. A comparison of their molecular weights with those of the databases suggested that the sera from allergic patients may react to four allergens: Pyr c 1 (18 kDa), Pyr c 3 (9 kDa), Pyr c 4 (14 kDa), and Pyr c 5 (34 kDa). Additionally, these patients showed immunoreactivity against six other proteins with MW ranging from 6–60 kDa, whose databases do not describe these proteins as allergens.

#### 3.3.2. Peach (*Prunus persica*)

The IgE-mediated reaction profile revealed that the serum of patients reacted with seven proteins. These immunogenic proteins had an MW ranging from 18 to 60 kDa (Figure 2C). Comparison with databases suggested that five immunogenic proteins matched the MW of known allergens: Pru p 1 or Pru p 9 (18 kDa), Pru p 2 (25 kDa) and Pru p 10 (46 kDa). Besides, a band of 60 kDa reacted with the IgE of patients, which matches with mandelonitrile lyase, a homolog of Pru du 10 of almond (*Prunus dulcis*) [32]. Additionally, the serum of patients reacts to three proteins of 30, and 49 kDa, which do not correspond to any peach allergens described in the databases.

#### 3.3.3. Banana (*Musa paradisiaca*)

The study showed that the pooled serum samples presented immunoreactivity against eight proteins with MW ranged from 9 to 59 kDa (Figure 2D). Comparison with the databases revealed six known allergens: Mus a 2 (34 kDa), Mus a 3 (9 kDa), Mus a 4 (20 kDa), Mus a 5 (36 kDa) and Mus at 6 (27 kDa), and Mus a 7 (catalase; 59 kDa) who was recently described as allergen in this fruit [33,34]. Besides, the sera reacted with two other proteins with MW of 19, and 44 kDa. These MWs do not match any allergens described in the databases.

#### 3.3.4. Avocado (*Persea americana*)

The case of a patient allergic to avocado is relevant, as anaphylaxis occurs within minutes after accidentally ingesting this food. In Mexico, avocado is eaten raw in the diet. The serum of the patient reacted with 11 proteins (Figure 2E), with MW ranging from 7 to 60 kDa. Databases comparison revealed two known allergens: Pers a 1 (35 kDa), and Pers a 4 (profilin, 13 kDa). Notably, four new potential allergens were identified in 2012 [24]; three bands of WB matched the MW of such proteins: thaumatin lipid protein (TLP, 27 kDa), glucanase (GLU, 30 kDa), and polygalacturonase (POL, 40 kDa). Therefore, it is evident that the patient reacted to six proteins whose molecular weights have not been previously described as allergens.

In addition, several identified allergens are also found in the pollens that sensitized our patients (Table 1). For instance, the pollen of the three species contains allergens from the PR-10 family, which are homologous to peach (Pru p 1) and pear (Pyr c 1) allergens. Remarkably, the assays conducted with samples from control patients confirmed the absence of nonspecific reactions for all the studied fruits.

### 3.4. Comparison of the Possible Identified Allergens Shared Among the Fruit Profiles

A comparison of the allergen profiles of the four fruits suggested that there is reactivity to homologous allergens present in more than one type of fruit (Table 2). We found a total of six families of allergenic proteins, which are broadly distributed among the four fruits. Thaumatin-like protein (TLP) was potentially found in three fruits: peach (Pru p 2), banana (Mus a 4) and avocado. Profilins were also found in three fruits: pear (Pyr c 4), banana (Mus a 1), and avocado. The other four families were found in two fruits. For instance, PR-10 proteins were observed in peach (Pru p 1) and pear (Pyr c 1); the presence of these proteins in WB assays correlates with the positive data of patients with specific IgE tests against the PR-10 (Table 1), supporting their precise identification.

In addition, we found patients with allergies to more than one type of fruit. This result correlates with the finding that several homologous allergens are shared among the fruits studied here (Table 2), which may contribute to the multiple reactivities in these foods.

## 4. Discussion

Fruits from the Rosaceae family (apple, pear, and peach) and the Musaceae family (banana) are frequently the first solid foods given to infants during the ablactation process. These foods are the primary allergens affecting the Mexican pediatric population [14]. Although these fruits are highly relevant allergens in Mexico, there is limited information on the molecular epidemiology of their allergenic proteins in this country. Furthermore, it is unknown whether the Mexican population exhibits allergenicity only against the proteins described in the current databases or whether there may be unidentified allergenic components with clinical significance. In this context, this work provides evidence that such a circumstance is indeed possible. For example, in pear, we found four proteins that matched the MW reported in the analyzed databases, whereas six did not match. In the peach, five proteins matched, whereas three did not. Six proteins matched with reported allergens in the banana, and two did not. Avocado was an extreme case; two bands matched with allergens described in databases, whereas another three bands matched with new potential allergens identified [24]; finally, six bands did not match with previously described allergens. Hence, we found 16 immunoreactive proteins with MW’s that do not correspond to any reported allergens. Our protein extraction technique probably enabled us to extract proteins with low expression abundance in these foods, resulting in many previously unreported immunogenic proteins. These findings strongly suggest the presence of potential new undescribed allergenic components in these foods, which encouraged us to continue with their characterization. Conversely, the inability to detect the remaining allergens reported by the allergen databases in these foods may be attributed to the small number of patients included in this study.

Most of the patients were diagnosed with PFAS and were sensitized to pollens from *Quercus*, *Alnus*, or *Betula* (Table 1). Accordingly, these pollens are the primary sensitizers containing allergens also identified in fruits. For instance, *Betula* contains allergens from the Phenylcoumaran benzylic ether reductase (Bet v 6) family, which is homologous with Pyr c 5 found in pear. *Betula* and *Quercus* contain profilins (Bet v 2 and Que ac 2, respectively), which are homologous to Pyr c 4 from pear, Mus a 1 from banana, and a profilin identified in avocado [24]. In addition, the pollen of the three species contains allergens from the PR-10 family, which are homologous to peach (Pru p 1) and pear (Pyr c 1) allergens. Moreover, we found patients with allergies to more than one type of fruit. This result correlates with the finding that several homologous allergens are shared among the fruits studied here (Table 2), which may contribute to the multiple reactivities in these foods.

A limitation of this work is the sample size, consisting of 16 patients and one adult. In this sense, the representativeness of the Mexican pediatric population was compromised. The global statistical power evaluation of the study showed a value of 17.6%; hence, the preliminary outcomes of this exploratory study must be interpreted with caution. Another significant limitation of this work is the inaccuracy of the determined molecular weights of the identified potential immunogenic proteins. A warning must be considered concerning the designation of suggested identified proteins, mainly in higher molecular weight, where the uncertainty is more significant.

In this context, the next step is to perform immunoproteomics for each one of the fruits to unambiguously identify immunoreactive proteins. This powerful and precise technique has proven effective in identifying novel allergens [27,31]. Through this approach, we recently identified nine potential allergenic components in apple (*Malus domestica*) that had not previously been described [27]. These components are currently being validated as allergens by constructing their recombinant forms. We are currently performing immunoproteomic analysis of all these fruits. In the case of fruits, mass spectrometry is the most appropriate tool for identification because the complete genome of these foods has been described [31]. However, Edman microdegradation for de novo sequencing may be the tool of choice for allergenic organisms where the genome is unavailable [35,36].

### Fruit Allergies in the Asia-Pacific Region

To assess a comparison among the Latin Ameria populations, we select the Asia-Pacific region for contrast with the description in this work. Notably, contrary to food allergy studies to seafood and peanuts, in which there are extensive publications showing significant knowledge about the most clinically relevant molecular components [37,38], fruit allergy studies are limited and are based primarily on self-reported surveys, parental surveys, or physician diagnoses without OFC testing. Consequently, the data reveal significant biases that must be considered [39]. Hence, the reported prevalence ranged from 0.03% to 8%, depending on the population and the clinical cases evaluated [40]. Like Europe and Latin America, fruit varieties in the Asia-Pacific zone depend on regional weather, exhibiting different patterns of allergies. For instance, allergies to fruits from the Rosaceae family are more frequent in Japan [41]. In contrast, fruit allergies caused by non-Rosaceae fruits are more relevant for the rest of the Asia countries. Kiwi and banana generate allergies mainly in Southeast Asia (Thailand, 2.1% prevalence) [42,43], South Asia (India, 0.5% prevalence) [44], and Southwest Asia (Iran, Israel, Turkey with 0.4, 3, and 1–7% prevalence, respectively) [45,46,47,48]. In contrast, mango allergy is more frequent in East Asia (China and Taiwan with 0.75–1.86% and 3.6% prevalence, respectively) [49,50,51,52].

Like Latin America, the clinical manifestations diversity in Asia-Pacific range from local symptoms to anaphylaxis; besides, food allergies are frequently caused by PFAS [39]. However, several decades ago, it was also identified that China, Japan, South Africa, and Australia exhibited a condition described as lipid transfer protein (LTP) syndrome; such an entity displays allergic reactions generated by cross-reactivity among two non-related LTP [53]. Furthermore, it has been reported that latex-fruit syndrome (LFS) is present, which involves cross-reactivity between latex and different fruits, such as avocado, banana, kiwi, and chestnut [54]. Class I Chitinase (Hev b 6) has been identified as the main responsible component of LFS [39].

Concerning component resolved diagnosis, the Asia-Pacific data is more abundant than in Mexico, but more limited than in Europe and the United States. For instance, in a study from Japan, Bet v 1 and profilin homologous were the primary sensitizers in cases, indicating a PFAS mechanism [55]. In contrast, Pru p 7 (gibberellin-regulated protein) from peach was the principal allergen in non-PFAS patients [41]. In China, the most relevant mango proteins were Man i 1 (Class IV Chitinase), Man i 2 (Bet v 1-related protein), and Man i 4 (profilin) [56].

Collectively, the Latin America and Asia-Pacific regions require to increase fruit allergy studies at the molecular level conducted to determine the most relevant clinical allergenic components, as well as to discover potential novel regional or global allergens. The relevance of obtaining new information about allergens is that these molecular data can be used to develop essential information about their clinical significance. This includes studies of their sensitization frequencies, diagnostic value, and associations with symptom severity, whether they are major allergens or panallergens that cause cross-reactivity, and the identification of new epitopes. This information is invaluable for achieving more accurate diagnoses and optimizing immunotherapy treatments. It enables precision medicine, which tailors medical practice by individually guiding therapeutic interventions.

## 5. Conclusions

The pollen-food allergy syndrome patients analyzed in this study presented IgE-specific activity against fruit proteins not reported in the WHO/IUIS databases. This suggests the presence of undescribed allergenic components. This research is significant because it will encourage immunoproteomic studies to identify immunoreactive proteins and discover novel allergens.

## Figures and Tables

**Table 1:** Clinical data of patients.

Allergic Disease ___ #Sample	Allergic Rhinitis	Oral Allergy Syndrome	Asthma	Atopic Dermatitis	Allergic Conjunctivitis	Anaphylaxisss	Challenge to Fruits						IgE Total (UI/mL) ± SD	Reactivity to Pollen Skin Prick Test			sIgE to Bet v1 (UI/A)
							Oral Food Challenge			Skin Prick Test (mm × mm)							
							Pear	Peach	Banana	Pear	Peach	Banana		*Quercus*	*Alnus*	*Betula*	
P1	(+)	(+)	(−)	(−)	(−)	(−)	(−)	(+)	(+)	NE	ND	ND	233.8 +/− 58.9	(+)	(+)	(+)	ND
P2	(+)	(+)	(+)	(+)	(−)	(−)	(−)	(−)	(+)	NE	NE	ND	63.9 +/− 22.7	(+)	(+)	(+)	ND
P3	(+)	(+)	(−)	(−)	(−)	(−)	(−)	(−)	(+)	ND	ND	5 × 10	126.7 +/− 24.0	(+)	(+)	(+)	ND
P4	(+)	(+)	(−)	(−)	(−)	(−)	(+)	(+)	(−)	ND	ND	NE	30.3 +/− 5.6	(+)	(−)	(+)	ND
P5	(+)	(−)	(−)	(−)	(−)	(−)	(−)	(+)	(−)	NE	ND	NE	12.9 +/− 3.0	Negative			ND
P6	(+)	(+)	(+)	(−)	(−)	(−)	(+)	(+)	(−)	10 × 12	6 × 5	NE	149.1 +/− 50.5	(+)	(−)	(+)	ND
P7	(+)	(+)	(−)	(−)	(−)	(−)	(+)	(+)	(−)	4 ×5	4 × 4	NE	16.1 +/− 5.41	(+)	(−)	(+)	5.8
P8	(+)	(+)	(−)	(−)	(−)	(−)	(+)	(+)	(−)	12 × 14	9 × 10	NE	31.6 +/− 32.0	(−)	(−)	(+)	3.44
P9	(+)	(+)	(+)	(−)	(−)	(−)	(−)	(+)	(−)	NE	12 × 12	NE	262.1 +/− 83.0	(+)	(−)	(+)	ND
P10	(+)	(+)	(+)	(+)	(−)	(−)	(−)	(−)	(+)	NE	NE	ND	218.3 +/− 29.2	(+)	(+)	(+)	ND
P11	(+)	(+)	(+)	(+)	(−)	(−)	(+)	(+)	(+)	ND	ND	ND	61.9 +/− 14.7	(+)	(+)	(−)	ND
P12	(+)	(−)	(−)	(+)	(−)	(−)	(−)	(+)	(−)	NE	ND	NE	325.1 +/− 52.0	Negative			ND
P13	(+)	(+)	(−)	(+)	(−)	(+)	(−)	(+)	(+)	NE	ND	ND	181.5 +/− 7.6	(+)	(+)	(−)	ND
P14	(+)	(+)	(−)	(+)	(−)	(−)	(−)	(+)	(−)	NE	12 × 13	NE	34.3 +/− 7.4	(+)	(+)	(+)	ND
P15	(+)	(+)	(+)	(−)	(−)	(−)	(−)	(+)	(−)	NE	ND	NE	120.3 +/− 46.6	Negative			ND
P16	(+)	(+)	(+)	(+)	(+)	(−)	(−)	(+)	(−)	NE	ND	NE	167.8 +/− 25.6	(+)	(−)	(−)	ND

NE = Not evaluated since OFC assay was negative, (+) = Positive result, sIgE: Specific IgE, (−) = Negative result, ND = Not done.

**Table 2:** Potential identified allergens shared among the fruits.

Protein	MW (kDa)	Peach (*Prunnus persica*)	Pear (*Pyrus communis*)	Banana (*Musa acuminata*)	Avocado (*Persea americana*)
Pathogenesis-related protein (PR-10)	17–18	Pru p 1	Pyr c 1	NO	NO
Thaumatin-like protein (TLP)	25–28	Pru p 2	NO	Mus a 4	ID
Profilin	13–15	NO	Pyr c 4	Mus a 1	ID
Beta-1, 3-Glucanase	30	NO	NO	Mus a 5	ID
Class I Chitinase	33–34	NO	NO	Mus a 2	Pers a 1
Polygalacturonase	40–45	Pru p 10	NO	NO	ID

NO. Non observed in this study. ID. Identified in this study.

## Data Availability

The original contributions presented in this study are included in the article. Further inquiries can be directed to the corresponding authors.

## Supplementary Materials

The following supporting information can be downloaded at: https://www.mdpi.com/article/10.3390/diseases13090284/s1, Figure S1: Original SDS-PAGE of fruit extracts; Figure S2: Original blots of (A) *Pyrus communis*; (B) *Prunnus persica* and *Musa paradisiaca*; (C) *Persea americana*; and (D) Control samples.

## Abbreviations

The following abbreviations are used in this manuscript:

WHO/IUIS	World Health Organization/International Union of Immunological Societies
PFAS	Pollen-Food Allergy Syndrome
OAS	Oral Allergy Syndrome
SPT	Skin Prick Test
WB	Western Blot
