# Correction: Torres-Arroyo et al. Immunoblotting Analysis of Fruit Proteins in Mexican Pediatric Patients Suggests the Existence of New Allergens. *Diseases* 2025, *13*, 284

**DOI:** 10.3390/diseases14010013

**Published:** 2025-12-30

**Authors:** Angélica Torres-Arroyo, Maidelen Suárez-Gutiérrez, Andrea Iglesias-Amaya, Aramiz López-Durán, Luisa Díaz-García, Horacio Reyes-Vivas, David Alejandro Mendoza-Hernández

**Affiliations:** 1 Laboratorio de Bioquímica-Genética, Instituto Nacional de Pediatría, Av. Insurgentes Sur No. 3700-C, Col. Insurgentes Cuicuilco, Alcaldía Coyoacán, Mexico City C.P. 04530, Mexico; angelica.tarroyo@gmail.com (A.T.-A.); maide15@hotmail.es (M.S.-G.); 2 División de Ciencias Biológicas y de la Salud, Universidad Autónoma Metropolitana, Campus Iztapalapa, Mexico City C.P. 09340, Mexico; 3 Servicio de Alergia, Instituto Nacional de Pediatría, Av. Insurgentes Sur No. 3700-C, Col. Insurgentes Cuicuilco, Alcaldía Coyoacán, Mexico City C.P. 04530, Mexico; andrea.unam95@gmail.com; 4 Servicio de Ortopedia, Instituto Nacional de Pediatría, Av. Insurgentes Sur No. 3700-C, Col. Insurgentes Cuicuilco, Alcaldía Coyoacán, Mexico City C.P. 04530, Mexico; aramizl@hotmail.com; 5 Departamento de Metodología de la Investigación, Instituto Nacional de Pediatría, Av. Insurgentes Sur No. 3700-C, Col. Insurgentes Cuicuilco, Alcaldía Coyoacán, Mexico City C.P. 04530, Mexico; luisadiazg@gmail.com

## Figure Legend

In the original publication, there was a mistake in the Supplementary Materials. The captions for Figures S3–S6 were incorrectly included in the published version [1]. The updated Supplementary Materials PDF (with these captions removed) appears below. 

Supplementary Materials: The following supporting information can be downloaded at: https://www.mdpi.com/article/10.3390/diseases13090284/s1, Figure S1: Original SDS-PAGE of fruit extracts; Figure S2: Original blots of (A) *Pyrus communis*; (B) *Prunnus persica* and *Musa paradisiaca*; (C) *Persea americana*; and (D) Control samples.

## Missing Institutional Review Board Statement and Informed Consent Statement

There was an error in the original publication [1]. The IRBS and ICS Sections were omitted from the back matter.

A correction has been made to the Back Matter, IRBS and ICS Sections:

The missing IRBS and ICS Sections have now been added. “Institutional Review Board Statement: The study was conducted following the Declaration of Helsinki and approved by Instituto Nacional de Pediatria (Registration No. INP-035/2018, approval date: 9 July 2018).”

“Informed Consent Statement: Written informed consent has been obtained from the patient(s) to publish this paper.”

The authors state that the scientific conclusions are unaffected. This correction was approved by the Academic Editor. The original publication has also been updated.
