# Supplementary material for: Immunoblotting Analysis of Fruit Proteins in Mexican Pediatric Patients Suggests the Existence of New Allergens
Source: Diseases. 2025 Sep 1;13(9):284. doi: 10.3390/diseases13090284 (PMC12468298; doi:10.3390/diseases13090284)
Supplement: Supplementary file 1 [file diseases-13-00284-s001.zip › diseases-3769699-supplementary.pdf]

SUPPLEMENTARY MATERIAL

Immunoblotting Analysis of Fruit Proteins in Mexican Pediatric Patients Suggests the Existence of New Allergens

Angélica Torres-Arroyo[1,2], Maidelen Suárez-Gutiérrez[1], Andrea Iglesias-Amaya[3], Aramiz López-Durán[4], Luisa Díaz-García[5], Horacio Reyes-Vivas[1]*, and David Alejandro Mendoza-Hernández[3]*

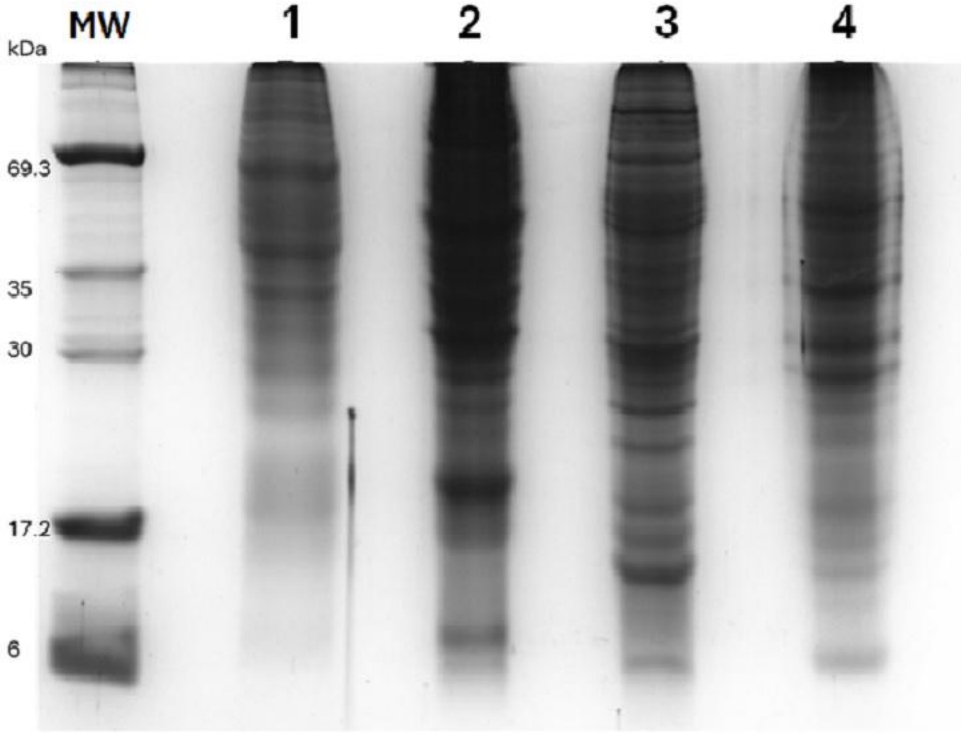

Figure S1. Original SDS-PAGE of fruit extracts

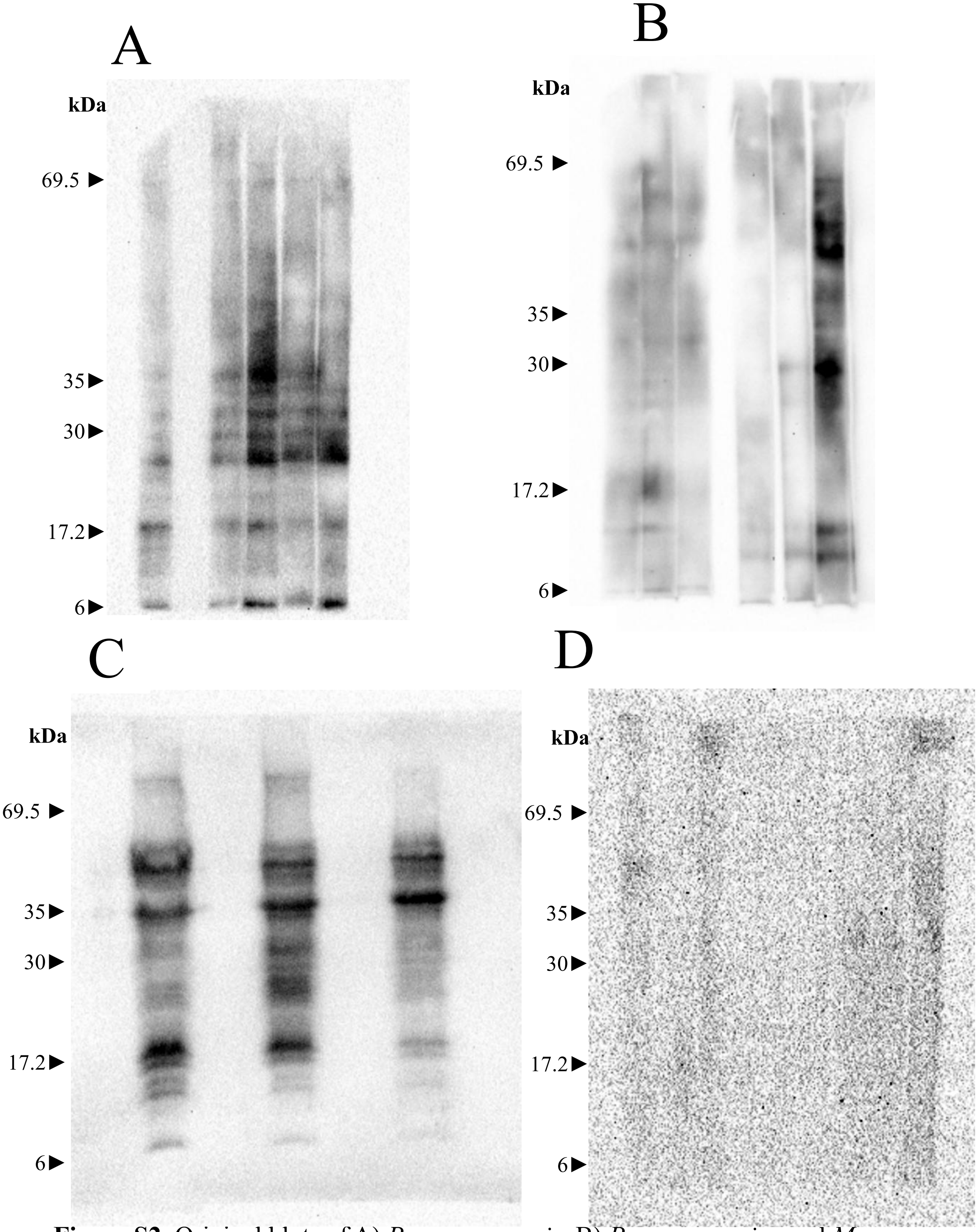

Figure S2. Original blots of A) *Pyrus communis*; B) *Prunnus persica* and *Musa paradisiaca*; C) *Persea americana*; and D) Control samples.

Protocolo No. 035/2018

INSTITUTO NACIONAL DE PEDIATRÍA
INP
09 JUL 2018
APROBADO
COMITÉ DE ÉTICA
EN INVESTIGACIÓN

Instituto Nacional de Pediatría

CONSENTIMIENTO INFORMADO DE LOS PADRES O TUTORES PARA QUE SU HIJO (A) PARTICIPEN EN EL ESTUDIO DE INVESTIGACION: **"ESTUDIO INMUNOPROTEÓMICO DE LOS ALÉRGENOS RELACIONADOS CON HIPERSENSIBILIDAD TIPO 1 A LAS FRUTAS EN PACIENTES PEDIÁTRICOS DEL INP. DESARROLLO DE UN KIT DIAGNÓSTICO, No. 035/2018"**

INVESTIGADOR PRINCIPAL: DR. HORACIO REYES VIVAS

PARTE I Información

Estimado Padre o Tutor:

Se le invita a su hijo(a) a participar en un estudio en el que se evaluaran las características de los pacientes con alergia a manzana, durazno, pera y/o plátano, mediante la aplicación de cuestionarios, así como determinación de unas moléculas en sangre denominadas inmunoglobulinas "E" que pueden reaccionar en extractos de fruta. Todos estos estudios se realizan en pacientes con diagnóstico de alergia a alguna de estas frutas para determinar si existen otras substancias además de las reportadas en la literatura, causantes de la sintomatología de la alergia a estas frutas y con esto aportar nuevo conocimiento acerca de las causas de esta enfermedad.

Lea cuidadosamente este formato y pregunte al médico del estudio cualquier duda al respecto.

La participación en esta investigación es voluntaria. Si permite la participación de su hijo(a) deberá dar por escrito su consentimiento, firmando este formato. Para hacerlo, es necesario leer la información contenida en este documento y aclarar todas sus dudas con el responsable del estudio. En caso contrario, que decida no participar, seguirá recibiendo su atención médica en esta Institución de manera regular, sin ningún cambio o diferencia en su trato.

3) ¿Para qué se efectúa este estudio?

Se desea obtener información para determinar si existen otras substancias además de las reportadas en la literatura causantes de los síntomas de la alergia a estas frutas y con esto aportar nuevo conocimiento acerca de las causas de esta patología. Esto nos permitirá obtener a mediano plazo un kit que apoye a los Doctores a diagnosticar más rápida y certeramente. Sin embargo, debido a que este kit se comenzará a desarrollar en algunos años, el paciente no podrá obtener beneficios de este desarrollo. Para este estudio, es necesario muestras de

pacientes que NO PRESENTEN diagnóstico de alergia; estas muestras se compararán contra aquellas muestras de pacientes que si fueron diagnosticados contra alergia alimentaria.

2) ¿Quiénes pueden participar en el estudio?

Pueden participar en el estudio todos los pacientes que NO PRESENTEN antecedentes alérgicos.

3) ¿Qué se le pedirá al paciente que haga en el estudio?

Después de que haya firmado el consentimiento informado, se incluirá a su hijo(a) en el estudio, por lo que la información proporcionada en la consulta, respecto a sus síntomas y antecedentes familiares se utilizarán en nuestra base de datos. Posteriormente, el personal médico tomará una muestra sanguínea de 5 mililitros, equivalentes a una cuchara normal, y se utilizará para los siguientes estudios:

- Estudios para detectar si existen en la sangre unas moléculas denominadas inmunoglobulinas tipo "E".
- Ensayos para conocer si las moléculas del paciente pueden identificar algunas substancias de las frutas.
- Finalmente, si las moléculas del paciente identifican algunas substancias en los frutos, trataremos de saber cuáles son los nombres de estas substancias en el fruto que se relacionan con los síntomas presentados en la alergia del paciente.

4) ¿Cuáles son los riesgos asociados o efectos indeseables que pueden pasarle a mi hijo(a) al participar en el estudio?

Los efectos que pueden presentarse al tomar una muestra de sangre son generalmente transitorios y con intensidad leve. Los eventos más frecuentes son dolor leve en la zona de punción y aparición de un moretón. En caso de que se presentará este problema y se requiriera de manejo hospitalario, el investigador principal y/o el equipo de investigación se encargará de todo lo necesario para su atención en esta Institución.

5) ¿Alguno de los procedimientos duele?

Para la toma de la muestra sanguínea, se realiza un piquete, el cual no suele ser más doloroso que la picadura de una hormiga.

6) ¿Qué beneficios obtiene mi hijo(a) del estudio?

INSTITUTO NACIONAL DE PEDIATRÍA
INP
09 JUL 2018
APROBADO
COMITÉ DE ÉTICA EN INVESTIGACIÓN

Todos los procedimientos que se realizarán son estudios que se necesitan para conocer más acerca de la alergia que provocan ciertas frutas (manzana, durazno, pera y plátano). Algunos datos nos pueden dar información acerca de la causa, de tal forma que en algunos casos, podemos ofrecer tratamiento para el mismo. Además que todos los estudios se realizarán de manera gratuita.

7) ¿A quién debe llamar si tengo más preguntas?

En dado caso que tuviera más preguntas después de leer esta información, antes o durante el estudio, podrá contactar siempre al responsable del estudio o a cualquier integrante del mismo (Dr. Horacio Reyes Vivas teléfono 10 84 0900, ext. 1442); así como al Presidente del Comité de Ética, (Dra. Matilde Ruiz García, teléfono 10 84 0900, ext. 1581), en caso de que tenga dudas sobre los derechos del participante.

8) ¿Quiénes van a tener información de los datos de mi hijo(a) datos?

La información se tratará en la forma más confidencial posible de conformidad con las leyes locales, estatales, federales e internacionales. La información de este estudio será del conocimiento del Instituto Nacional de Pediatría. Su información es confidencial, los datos recolectados para el estudio, serán revisados por personas autorizadas por el centro de estudio. Esto es con el fin de garantizar que el estudio se llevó a cabo de acuerdo con las normas de calidad de las Buenas Prácticas Clínicas.

9) ¿Puedo conocer los resultados del estudio?

Sí, la intención es obtener un diagnóstico certero del síndrome de alergia a las frutas (manzana, pera, durazno o plátano), de tal forma que ustedes puedan conocer si alguno de los mencionados alimentos realmente se asocian a los síntomas que presenta su hijo(a).

10) ¿Tiene algún costo el estudio?

No, como ya se mencionó, todos los estudios serán totalmente gratuitos.

INSTITUTO NACIC
DE PEDIATRÍA
INP
09 JUL 2018
APROBADO
COMITÉ DE ÉTICA
EN INVESTIGACIÓN

DECLARACIÓN DEL PADRE O TUTOR

He leído y entiendo el presente consentimiento, mis preguntas han sido contestadas y acepto participar en este estudio.

Doy, voluntariamente, mi consentimiento para que mi hijo (a) participe en este estudio de investigación, hasta que yo decida lo contrario. Entiendo que mi participación es voluntaria y que puedo retirarme en cualquier momento, sin dar ninguna razón y sin que los derechos de mi hijo (a) se vean afectados.

Entiendo que las secciones relevantes de cualquiera de las notas y datos médicos recabados durante el estudio pueden ser examinados. Permito que el investigador y sub-investigador del estudio, tengan acceso a la información que derive del estudio.

Voy a recibir un duplicado firmado de esta forma de consentimiento.

CONSENTIMIENTO INFORMADO

Fecha (dd-mm-aaaa): ____________________

Nombre del paciente:

____

Nombre del tutor o representante legal:

____

Firma del tutor o representante legal:

____ Domicilio:

____

DECLARACIÓN DE LOS TESTIGOS

La información en esta forma de consentimiento informado se explicó al participante quien al parecer la comprendió.

Nombre (1er testigo):

____ Relación con el paciente:

____

Firma del primer testigo:

____

Fecha (dd-mmm-aaaa):

____

Domicilio:

INP INSTITUTO NACIONAL DE PEDIATRÍA
09 JUL 2018
APROBADO
COMITÉ DE ÉTICA
EN INVESTIGACIÓN

____

Nombre (2o testigo):

____ Relación con el paciente:

____

Firma del primer testigo:

____ Fecha (dd-mmm-aaaa):

____

Domicilio:

____

DECLARACIÓN DEL PERSONAL DEL ESTUDIO

La persona o personas que firman esta forma de consentimiento informado recibieron una explicación completa y correcta y se les dio la oportunidad de hacer preguntas con relación a la naturaleza, riesgos y beneficios de la participación del paciente en esta investigación.

Nombre de la persona que obtiene el consentimiento:

____

Firma de la persona que obtiene el consentimiento:

____

Cargo en el estudio:

____

Fecha (dd-mmm-aaaa): ____________________________

INSTITUTO NACIONAL DE PEDIATRÍA
INP
09 JUL 2018
APROBADO
COMITÉ DE ÉTICA EN INVESTIGACIÓN

Figure S3. Letter of consent for controls.

No. Protocolo: 035/2018

INP INSTITUTO NACIONAL DE PEDIATRÍA
09 JUL 2018
APROBADO
COMITÉ DE ÉTICA
EN INVESTIGACIÓN

ASENTIMIENTO INFORMADO PARA QUE EL PACIENTE PARTICIPE EN EL ESTUDIO DE INVESTIGACIÓN: **"ESTUDIO INMUNOPROTEÓMICO DE LOS ALÉRGENOS RELACIONADOS CON HIPERSENSIBILIDAD TIPO 1 A LAS FRUTAS EN PACIENTES PEDIÁTRICOS DEL INP. DESARROLLO DE UN KIT DIAGNÓSTICO, No. 035/2018"**

INVESTIGADOR PRINCIPAL: DR. HORACIO REYES VIVAS

PARTE I Información

Estimado Paciente:

Se te invita a participar en un estudio donde se evaluarán las características de los pacientes con alergia a manzana, durazno, pera y/o plátano, mediante la aplicación de cuestionarios, así como el análisis de una muestra de sangre. Todos estos estudios se realizan en pacientes con diagnóstico de alergia a alguna de estas frutas para conocer qué posibles substancias en la fruta causan los síntomas de alergia. Con lo anterior, se aportará nuevo conocimiento acerca de estas enfermedades.

Por favor, lee cuidadosamente este formato y pregunta a tu médico cualquier duda sobre el estudio.

La participación en esta investigación es voluntaria. Si decides participar deberás darnos por escrito tu consentimiento firmando este formato. Para hacerlo, es necesario que leas cuidadosamente la información contenida en este documento y aclarar todas tus dudas con el responsable del estudio. En el caso contrario que decidas no participar, seguirás recibiendo atención médica en esta Institución de manera regular, sin ningún cambio o diferencia en su trato.

1) ¿Para qué se efectúa este estudio?

Se desea obtener información para conocer si existen otras substancias en los frutos que causen la alergia, además de las reportadas en la literatura. Con esto, se aportará nuevo conocimiento acerca de estas enfermedades. Para este estudio, se requieren de muestras de pacientes que NO PRESENTEN diagnóstico de alergias; estas muestras se compararán contra aquellas muestras de pacientes que si fueron diagnosticados con alergia alimentaria.

2) ¿Quiénes pueden participar en el estudio?

Pueden participar en el estudio todos los pacientes que NO PRESENTEN antecedentes alérgicos.

3) ¿Qué se me pedirá que haga en el estudio?
Después de haber firmado el consentimiento informado, se te incluirá en el estudio, por lo que la información proporcionada en la consulta respecto a tus

síntomas y antecedentes familiares se almacenarán en nuestra base de datos. Posteriormente, se te solicitará que nos permitas tomarte una muestra de sangre de 5 mililitros, equivalentes a una cuchara, mediante la punción con una jeringa nueva y estéril. Será el personal médico el que se encargue de tomarte la muestra de sangre. Con tu muestra haremos los siguientes estudios:

- Un primer estudio para saber si en tu sangre tienes unas moléculas llamadas inmunoglobulinas tipo "E".
- Si estas moléculas están presentes en tu sangre, entonces analizaremos si reaccionan con las frutas.
- Finalmente, si tu sangre reacciona con las frutas, trataremos de conocer a qué parte de la fruta reaccionan tus moléculas.

4) ¿Cuáles son los riesgos asociados o efectos indeseables que pueden pasarme al participar en el estudio?

Los eventos que pueden presentarse al tomar una muestra de sangre son generalmente momentáneos y de intensidad leve. Los eventos más frecuentes son: dolor leve en la zona de punción y aparición de un moretón. En caso de que se presentara este problema y se requiriera de manejo hospitalario, el investigador principal y/o el equipo de investigación se encargarán de todo lo necesario para atenderte en esta Institución.

5) ¿Alguno de los procedimientos duele?

Para la toma de la muestra sanguínea, se realiza un piquete, el cual no suele ser más doloroso que la picadura de una hormiga. La obtención de sangre lo realizan personal con experiencia en esos métodos.

6) ¿Qué beneficios obtengo del estudio?

Todos los procedimientos que se realizarán son estudios que se necesitan para conocer más acerca de la alergia que provocan algunas frutas (manzana, durazno, pera y plátano). Se podrá obtener información acerca de la causa que produce la alergia. Por tanto, en algunos casos podemos ofrecer tratamiento para el mismo. Además, estos estudios se realizarán de manera gratuita.

7) ¿A quién debes llamar si tengo más preguntas?

En dado caso que tuviera más preguntas después de leer esta información, antes o durante el estudio, podrás contactar siempre al responsable del estudio o a cualquier integrante del mismo (Dr. Horacio Reyes Vivas teléfono 10 84 0900, ext.

INP INSTITUTO NACIONAL DE PEDIATRÍA
19 JUL 2018
APROBADO
COMITÉ DE ÉTICA EN INVESTIGACIÓN

1442); así como al Presidente del Comité de Ética, (Dra. Matilde Ruiz García, teléfono 10 84 0900, ext. 1581), en caso de que tenga dudas sobre los derechos del participante.

8) ¿Quiénes van a tener información de mis datos?

La información se tratará en la forma más confidencial posible de conformidad con las leyes locales, estatales, federales e internacionales. La información de este estudio será del conocimiento del Instituto Nacional de Pediatría. Su información es confidencial, los datos recolectados para el estudio se revisarán por personal autorizado por el centro de estudio. Esto es con el fin de garantizar que el estudio se llevó a cabo de acuerdo con las normas de calidad de las Buenas Prácticas Clínicas.

9) ¿Puedo conocer los resultados del estudio?

Sí, puedes obtener tu diagnóstico certero del síndrome de alergia a la fruta (manzana, pera, plátano o durazno). Por lo tanto, podrás conocer de los mencionados alimentos que parte se asocia a tus síntomas.

10) ¿Tiene algún costo el estudio?

No, como ya se señaló, todos los estudios serán totalmente gratuitos.

DECLARACIÓN DEL PARTICIPANTE

He leído y entiendo el presente consentimiento, mis preguntas han sido contestadas y acepto participar en este estudio.

Doy voluntariamente mi consentimiento para participar en este estudio de investigación, hasta que yo decida lo contrario. Entiendo que mi participación es voluntaria y que puedo retirarme en cualquier momento, sin señalar razón alguna y sin que mis derechos se vean afectados.

Entiendo que las secciones relevantes de cualquiera de las notas y datos médicos recabados durante el estudio pueden ser examinados. Permito que el investigador y sub-investigador del estudio, tengan acceso a la información que derive del estudio.

Voy a recibir un duplicado firmado de esta forma de consentimiento.

INSTITUTO NACIONAL DE PEDIATRÍA
INP
09 JUL 2018
APROBADO
COMITÉ DE ÉTICA EN INVESTIGACIÓN

No. Protocolo: 035/2018

Instituto
Nacional
de Pediatría

ASENTIMIENTO INFORMADO

Fecha (dd-mm-aaaa): ____________________

Nombre del paciente:

____

Nombre del tutor o representante legal:

____

Firma del tutor o representante legal:

____Domicilio:

____

DECLARACIÓN DE LOS TESTIGOS

La información en esta forma de consentimiento informado se explicó al participante quien al parecer la comprendió.

Nombre (1er testigo):

____ Relación con el paciente:

____

Firma del primer testigo:

____

Fecha (dd-mmm-aaaa):

____

Domicilio:

____

Nombre (2o testigo):

____ Relación con el paciente:

____

INSTITUTO NACIONAL DE PEDIATRÍA
INP
APROBADO
COMITÉ DE ÉTICA EN INVESTIGACIÓN
09 JUL 2018

No. Protocolo: 035/2018

Instituto
Nacional
de Pediatría

Firma del primer testigo:

____ Fecha (dd-mmm-aaaa):

____

Domicilio:

____

DECLARACIÓN DEL PERSONAL DEL ESTUDIO

La persona o personas que firman esta forma de consentimiento informado recibieron una explicación completa y correcta y se les dio la oportunidad de hacer preguntas con relación a la naturaleza, riesgos y beneficios de la participación del paciente en esta investigación.

Nombre de la persona que obtiene el consentimiento:

____

Firma de la persona que obtiene el consentimiento:

____

Cargo en el estudio:

____

Fecha (dd-mmm-aaaa): ________________________________

INSTITUTO NACIONAL
DE PEDIATRÍA
INP
09 JUL 2018
APROBADO
COMITÉ DE ÉTICA
EN INVESTIGACIÓN

Figure S4: Letter of informed assent for controls.

Protocolo No. 035/2018

INSTITUTO NACIONAL DE PEDIATRÍA
INP
09 JUL 2018
APROBADO
COMITÉ DE ÉTICA EN INVESTIGACIÓN

Instituto Nacional de Pediatría

CONSENTIMIENTO INFORMADO DE LOS PADRES O TUTORES PARA QUE SU HIJO (A) PARTICIPEN EN EL ESTUDIO DE INVESTIGACION: **"ESTUDIO INMUNOPROTEÓMICO DE LOS ALÉRGENOS RELACIONADOS CON HIPERSENSIBILIDAD TIPO 1 A LAS FRUTAS EN PACIENTES PEDIÁTRICOS DEL INP. DESARROLLO DE UN KIT DIAGNÓSTICO, No. 035/2018"**

INVESTIGADOR PRINCIPAL: DR. HORACIO REYES VIVAS

PARTE I Información

Estimado Padre o Tutor:

Se le invita a su hijo(a) a participar en un estudio en el que se evaluaran las características de los pacientes con alergia a manzana, durazno, pera y/o plátano, mediante la aplicación de cuestionarios, así como determinación de unas moléculas en sangre denominadas inmunoglobulinas "E" que pueden reaccionar en extractos de fruta. Todos estos estudios se realizan en pacientes con diagnóstico de alergia a alguna de estas frutas para determinar si existen otras substancias además de las reportadas en la literatura, causantes de la sintomatología de la alergia a estas frutas y con esto aportar nuevo conocimiento acerca de las causas de esta enfermedad.

Lea cuidadosamente este formato y pregunte al médico del estudio cualquier duda al respecto.

La participación en esta investigación es voluntaria. Si permite la participación de su hijo(a) deberá dar por escrito su consentimiento, firmando este formato. Para hacerlo, es necesario leer la información contenida en este documento y aclarar todas sus dudas con el responsable del estudio. En caso contrario, que decida no participar, seguirá recibiendo su atención médica en esta Institución de manera regular, sin ningún cambio o diferencia en su trato.

3) ¿Para qué se efectúa este estudio?

Se desea obtener información para determinar si existen otras substancias además de las reportadas en la literatura causantes de los síntomas de la alergia a estas frutas y con esto aportar nuevo conocimiento acerca de las causas de esta patología. Esto nos permitirá obtener a mediano plazo un kit que apoye a los Doctores a diagnosticar más rápida y certeramente. Sin embargo, debido a que este kit se comenzará a desarrollar en algunos años, el paciente no podrá obtener beneficios de este desarrollo.

2) ¿Quiénes pueden participar en el estudio?

Pueden participar en el estudio todos los pacientes con diagnóstico de alergia a alguna de las siguientes frutas: manzana, pera durazno y plátano que acuden a la consulta externa del servicio de Alergia del Instituto Nacional de Pediatría.

3) ¿Qué se le pedirá al paciente que haga en el estudio?

INSTITUTO NACIONAL DE PEDIATRÍA INP 09 JUL 2018 APROBADO COMITÉ DE ÉTICA EN INVESTIGACIÓN

Después de que haya firmado el consentimiento informado, se incluirá a su hijo(a) en el estudio, por lo que la información proporcionada en la consulta, respecto a sus síntomas y antecedentes familiares se utilizarán en nuestra base de datos. Posteriormente, el personal médico tomará una muestra sanguínea de 5 mililitros, equivalentes a una cuchara normal, y se utilizará para los siguientes estudios:

- Estudios para detectar si existen en la sangre unas moléculas denominadas inmunoglobulinas tipo "E".
- Ensayos para conocer si las moléculas del paciente pueden identificar algunas substancias de las frutas.
- Finalmente, si las moléculas del paciente identifican algunas substancias en los frutos, trataremos de saber cuáles son los nombres de estas substancias en el fruto que se relacionan con los síntomas presentados en la alergia del paciente.

4) ¿Cuáles son los riesgos asociados o efectos indeseables que pueden pasarle a mi hijo(a) al participar en el estudio?

Los efectos que pueden presentarse al tomar una muestra de sangre son generalmente transitorios y con intensidad leve. Los eventos más frecuentes son dolor leve en la zona de punción y aparición de un moretón. En caso de que se presentará este problema y se requiriera de manejo hospitalario, el investigador principal y/o el equipo de investigación se encargará de todo lo necesario para su atención en esta Institución.

5) ¿Alguno de los procedimientos duele?

Para la toma de la muestra sanguínea, se realiza un piquete, el cual no suele ser más doloroso que la picadura de una hormiga.

6) ¿Qué beneficios obtiene mi hijo(a) del estudio?

Todos los procedimientos que se realizarán son estudios que se necesitan para conocer más acerca de la alergia que provocan ciertas frutas (manzana, durazno, pera y plátano). Algunos datos nos pueden dar información acerca de la causa, de tal forma que en algunos casos, podemos ofrecer tratamiento para el mismo. Además que todos los estudios se realizarán de manera gratuita.

INSTITUTO NACIONAL DE PEDIATRÍA
INP
09 JUL 2018
APROBADO
COMITÉ DE ÉTICA
EN INVESTIGACIÓN

7) ¿A quién debe llamar si tengo más preguntas?

En dado caso que tuviera más preguntas después de leer esta información, antes o durante el estudio, podrá contactar siempre al responsable del estudio o a cualquier integrante del mismo (Dr. Horacio Reyes Vivas teléfono 10 84 0900, ext. 1442); así como al Presidente del Comité de Ética, (Dra. Matilde Ruiz García, teléfono 10 84 0900, ext. 1581), en caso de que tenga dudas sobre los derechos del participante.

8) ¿Quiénes van a tener información de los datos de mi hijo(a) datos?

La información se tratará en la forma más confidencial posible de conformidad con las leyes locales, estatales, federales e internacionales. La información de este estudio será del conocimiento del Instituto Nacional de Pediatría. Su información es confidencial, los datos recolectados para el estudio, serán revisados por personas autorizadas por el centro de estudio. Esto es con el fin de garantizar que el estudio se llevó a cabo de acuerdo con las normas de calidad de las Buenas Prácticas Clínicas.

9) ¿Puedo conocer los resultados del estudio?

Sí, la intención es obtener un diagnóstico certero del síndrome de alergia a las frutas (manzana, pera, durazno o plátano), de tal forma que ustedes puedan conocer si alguno de los mencionados alimentos realmente se asocian a los síntomas que presenta su hijo(a).

10) ¿Tiene algún costo el estudio?

No, como ya se mencionó, todos los estudios serán totalmente gratuitos.

DECLARACIÓN DEL PADRE O TUTOR

He leído y entiendo el presente consentimiento, mis preguntas han sido contestadas y acepto participar en este estudio.

Doy, voluntariamente, mi consentimiento para que mi hijo (a) participe en este estudio de investigación, hasta que yo decida lo contrario. Entiendo que mi participación es voluntaria y que puedo retirarme en cualquier momento, sin dar ninguna razón y sin que los derechos de mi hijo (a) se vean afectados.

Entiendo que las secciones relevantes de cualquiera de las notas y datos médicos recabados durante el estudio pueden ser examinados. Permito que el investigador y sub-investigador del estudio, tengan acceso a la información que derive del estudio.

Voy a recibir un duplicado firmado de esta forma de consentimiento.

CONSENTIMIENTO INFORMADO

Fecha (dd-mm-aaaa): ____________________

Nombre del paciente:

____

Nombre del tutor o representante legal:

____

Firma del tutor o representante legal:

____Domicilio:

____

DECLARACIÓN DE LOS TESTIGOS

La información en esta forma de consentimiento informado se explicó al participante quien al parecer la comprendió.

Nombre (1er testigo):

____ Relación con el paciente:

____

Firma del primer testigo:

____

Fecha (dd-mmm-aaaa):

____

Domicilio:

____

Nombre (2o testigo):

INSTITUTO NACIONAL DE PEDIATRÍA INP
09 JUL 2018
APROBADO COMITÉ DE ÉTICA EN INVESTIGACIÓN

Protocolo No. 035/2018

Instituto Nacional de Pediatría

INP

_______________________________ Relación con el paciente:

____

Firma del primer testigo:

_______________________________ Fecha (dd-mmm-aaaa):

____

Domicilio:

____

DECLARACIÓN DEL PERSONAL DEL ESTUDIO

La persona o personas que firman esta forma de consentimiento informado recibieron una explicación completa y correcta y se les dio la oportunidad de hacer preguntas con relación a la naturaleza, riesgos y beneficios de la participación del paciente en esta investigación.

Nombre de la persona que obtiene el consentimiento:

____

Firma de la persona que obtiene el consentimiento:

____

Cargo en el estudio:

____

Fecha (dd-mmm-aaaa): _______________________________

INSTITUTO NACIONAL DE PEDIATRÍA
INP
09 JUL 2018
APROBADO
COMITÉ DE ÉTICA EN INVESTIGACIÓN

Figure S5. Letter of consent for allergic patients.

No. Protocolo: 035/2018

INSTITUTO NACIONAL DE PEDIATRÍA
INP
09 JUL 2018
APROBADO
COMITÉ DE ÉTICA
EN INVESTIGACIÓN

INP Instituto Nacional de Pediatría

ASENTIMIENTO INFORMADO PARA QUE EL PACIENTE PARTICIPE EN EL ESTUDIO DE INVESTIGACIÓN: **"ESTUDIO INMUNOPROTEÓMICO DE LOS ALÉRGENOS RELACIONADOS CON HIPERSENSIBILIDAD TIPO 1 A LAS FRUTAS EN PACIENTES PEDIÁTRICOS DEL INP. DESARROLLO DE UN KIT DIAGNÓSTICO, No. 035/2018"**

INVESTIGADOR PRINCIPAL: DR. HORACIO REYES VIVAS

PARTE I Información
Estimado Paciente:

Se te invita a participar en un estudio donde se evaluarán las características de los pacientes con alergia a manzana, durazno, pera y/o plátano, mediante la aplicación de cuestionarios, así como el análisis de una muestra de sangre. Todos estos estudios se realizan en pacientes con diagnóstico de alergia a alguna de estas frutas para conocer qué posibles substancias en la fruta causan los síntomas de alergia. Con lo anterior, se aportará nuevo conocimiento acerca de estas enfermedades.

Por favor, lee cuidadosamente este formato y pregunta a tu médico cualquier duda sobre el estudio.

La participación en esta investigación es voluntaria. Si decides participar deberás darnos por escrito tu consentimiento firmando este formato. Para hacerlo, es necesario que leas cuidadosamente la información contenida en este documento y aclarar todas tus dudas con el responsable del estudio. En el caso contrario que decidas no participar, seguirás recibiendo atención médica en esta Institución de manera regular, sin ningún cambio o diferencia en su trato.

1) ¿Para qué se efectúa este estudio?

Se desea obtener información para conocer si existen otras substancias en los frutos que causen la alergia, además de las reportadas en la literatura. Con esto, se aportará nuevo conocimiento acerca de estas enfermedades.

2) ¿Quiénes pueden participar en el estudio?

Pueden participar en el estudio todos los pacientes con diagnóstico de alergia a alguna de las siguientes frutas: manzana, pera, durazno y plátano que acuden a la consulta externa del servicio de la Alergia del Instituto Nacional de Pediatría.

3) ¿Qué se me pedirá que haga en el estudio? Después de haber firmado el consentimiento informado, se te incluirá en el estudio, por lo que la información proporcionada en la consulta respecto a tus síntomas y antecedentes familiares se almacenarán en nuestra base de datos. Posteriormente, se te solicitará que nos permitas tomarte una

- muestra de sangre de 5 mililitros, equivalentes a una cuchara, mediante la punción con una jeringa nueva y estéril. Será el personal médico el que se encargue de tomarte la muestra de sangre. Con tu muestra haremos los siguientes estudios:

- Un primer estudio para saber si en tu sangre tienes unas moléculas llamadas inmunoglobulinas tipo "E".

- Si estas moléculas están presentes en tu sangre, entonces analizaremos si reaccionan con las frutas.

- Finalmente, si tu sangre reacciona con las frutas, trataremos de conocer a qué parte de la fruta reaccionan tus moléculas.

4) ¿Cuáles son los riesgos asociados o efectos indeseables que pueden pasarme al participar en el estudio?

Los eventos que pueden presentarse al tomar una muestra de sangre son generalmente momentáneos y de intensidad leve. Los eventos más frecuentes son: dolor leve en la zona de punción y aparición de un moretón. En caso de que se presentara este problema y se requiriera de manejo hospitalario, el investigador principal y/o el equipo de investigación se encargarán de todo lo necesario para atenderte en esta Institución.

5) ¿Alguno de los procedimientos duele?

Para la toma de la muestra sanguínea, se realiza un piquete, el cual no suele ser más doloroso que la picadura de una hormiga. La obtención de sangre lo realizan personal con experiencia en esos métodos.

6) ¿Qué beneficios obtengo del estudio?

Todos los procedimientos que se realizarán son estudios que se necesitan para conocer más acerca de la alergia que provocan algunas frutas (manzana, durazno, pera y plátano). Se podrá obtener información acerca de la causa que produce la alergia. Por tanto, en algunos casos podemos ofrecer tratamiento para el mismo. Además, estos estudios se realizarán de manera gratuita.

7) ¿A quién debes llamar si tengo más preguntas?

En dado caso que tuviera más preguntas después de leer esta información, antes o durante el estudio, podrás contactar siempre al responsable del estudio o a cualquier integrante del mismo (Dr. Horacio Reyes Vivas teléfono 10 84 0900, ext.

INSTITUTO NACIONAL DE PEDIATRÍA
INP
09 JUL 2018
APROBADO
COMITÉ DE ÉTICA EN INVESTIGACIÓN

1442); así como al Presidente del Comité de Ética, (Dra. Matilde Ruiz García, teléfono 10 84 0900, ext. 1581), en caso de que tenga dudas sobre los derechos del participante.

8) ¿Quiénes van a tener información de mis datos?

La información se tratará en la forma más confidencial posible de conformidad con las leyes locales, estatales, federales e internacionales. La información de este estudio será del conocimiento del Instituto Nacional de Pediatría. Su información es confidencial, los datos recolectados para el estudio se revisarán por personal autorizado por el centro de estudio. Esto es con el fin de garantizar que el estudio se llevó a cabo de acuerdo con las normas de calidad de las Buenas Prácticas Clínicas.

9) ¿Puedo conocer los resultados del estudio?

Sí, puedes obtener tu diagnóstico certero del síndrome de alergia a la fruta (manzana, pera, plátano o durazno). Por lo tanto, podrás conocer de los mencionados alimentos que parte se asocia a tus síntomas.

10) ¿Tiene algún costo el estudio?

No, como ya se señaló, todos los estudios serán totalmente gratuitos.

DECLARACIÓN DEL PARTICIPANTE

He leído y entiendo el presente consentimiento, mis preguntas han sido contestadas y acepto participar en este estudio.

Doy voluntariamente mi consentimiento para participar en este estudio de investigación, hasta que yo decida lo contrario. Entiendo que mi participación es voluntaria y que puedo retirarme en cualquier momento, sin señalar razón alguna y sin que mis derechos se vean afectados.

Entiendo que las secciones relevantes de cualquiera de las notas y datos médicos recabados durante el estudio pueden ser examinados. Permito que el investigador y sub-investigador del estudio, tengan acceso a la información que derive del estudio.

Voy a recibir un duplicado firmado de esta forma de consentimiento.

INSTITUTO NACIONAL DE PEDIATRÍA
INP
09 JUL 2018
APROBADO
COMITÉ DE ÉTICA EN INVESTIGACIÓN

No. Protocolo: 035/2018

Instituto
Nacional
de Pediatría

ASENTIMIENTO INFORMADO

Fecha (dd-mm-aaaa): ____________________

Nombre del paciente:

____

Nombre del tutor o representante legal:

____

Firma del tutor o representante legal:

____ Domicilio:

____

DECLARACIÓN DE LOS TESTIGOS

La información en esta forma de consentimiento informado se explicó al participante quien al parecer la comprendió.

Nombre (1er testigo):

____ Relación con el paciente:

____

Firma del primer testigo:

____

Fecha (dd-mmm-aaaa):

____

Domicilio:

____

Nombre (2o testigo):

____ Relación con el paciente:

____

INSTITUTO NACIONAL DE PEDIATRÍA
INP
09 JUL 2018
APROBADO
COMITÉ DE ÉTICA EN INVESTIGACIÓN

No. Protocolo: 035/2018

Instituto Nacional de Pediatria

Firma del primer testigo:

____ Fecha (dd-mmm-aaaa):

____

Domicilio:

____

DECLARACIÓN DEL PERSONAL DEL ESTUDIO

La persona o personas que firman esta forma de consentimiento informado recibieron una explicación completa y correcta y se les dio la oportunidad de hacer preguntas con relación a la naturaleza, riesgos y beneficios de la participación del paciente en esta investigación.

Nombre de la persona que obtiene el consentimiento:

____

Firma de la persona que obtiene el consentimiento:

____

Cargo en el estudio:

____

Fecha (dd-mmm-aaaa): _____________________________

INSTITUTO NACIONAL DE PEDIATRÍA
INP
09 JUL 2018
APROBADO
COMITÉ DE ÉTICA EN INVESTIGACIÓN

Figure S6. Letter of informed assent for allergic patients.
